# Ensuring cross-cultural data comparability by means of anchoring vignettes in heterogeneous refugee samples

**DOI:** 10.1186/s12874-023-02015-2

**Published:** 2023-09-28

**Authors:** Natalja Menold, Louise Biddle, Hagen von Hermanni, Jasmin Kadel, Kayvan Bozorgmehr

**Affiliations:** 1 Dept. of Methods in Empirical Social Research, Technische Universität Dresden, Dresden, Germany; 2 Charité - Universitätsmedizin Berlin, Institute of International Health, Berlin, Germany; 3 University Hospital Heidelberg, Section for Health Equity Studies & Migration, Heidelberg, Germany; 4 Dept. of Population Medicine and Health Services Research, Bielefeld University, Bielefeld, Germany

**Keywords:** Comparative statistics, Measurement invariance, Response category related differential item functioning, Anchoring vignettes, Health system responsiveness, Sample heterogeneity, Refugees

## Abstract

**Background:**

Configural, metric, and scalar measurement invariance have been indicators of bias-free statistical cross-group comparisons, although they are difficult to verify in the data. Low comparability of translated questionnaires or the different understanding of response formats by respondents might lead to rejection of measurement invariance and point to comparability bias in multi-language surveys. Anchoring vignettes have been proposed as a method to control for the different understanding of response categories by respondents (the latter is referred to as differential item functioning related to response categories or rating scales: RC-DIF). We evaluate the question whether the cross-cultural comparability of data can be assured by means of anchoring vignettes or by considering socio-demographic heterogeneity as an alternative approach.

**Methods:**

We used the Health System Responsiveness (HSR) questionnaire and collected survey data in English (*n* = 183) and Arabic (*n* = 121) in a random sample of refugees in the third largest German federal state. We conducted multiple-group Confirmatory Factor Analyses (MGCFA) to analyse measurement invariance and compared the results when 1) using rescaled data on the basis of anchoring vignettes (non-parametric approach), 2) including information on RC-DIF from the analyses with anchoring vignettes as covariates (parametric approach) and 3) including socio-demographic covariates.

**Results:**

For the HSR, every level of measurement invariance between the Arabic and English languages was rejected. Implementing rescaling or modelling on the basis of anchoring vignettes provided superior results over the initial MGCFA analysis, since configural, metric and – for ordered categorical analyses—scalar invariance could not be rejected. A consideration of socio-demographic variables did not show such an improvement.

**Conclusions:**

Surveys may consider anchoring vignettes as a method to assess cross-cultural comparability of data, whereas socio-demographic variables cannot be used to improve data comparability as a standalone method. More research on the efficient implementation of anchoring vignettes and further development of methods to incorporate them when modelling measurement invariance is needed.

**Supplementary Information:**

The online version contains supplementary material available at 10.1186/s12874-023-02015-2.

## Introduction

Cross-cultural social science, as well as comparative psychological, educational, economic and health research has had a longstanding interest in comparisons of persons’ characteristics across or within countries and different ethnic and language subgroups. Self-reports in surveys have been a relevant data collection method. Increasing globalization, different political systems, religious conflicts, war and poverty mean that migration and refugee flows are now and will continue in the future to be one of the main human challenges facing societies endeavouring to integrate refugees through their participation in everyday life. A crucial part of this would be survey research to elicit refugees’ behaviour and opinions. 

Since the early days of comparative research, ensuring cross-language comparability of data, for example by means of appropriate translations and appropriate questionnaire design, has been recognized as a fundamental methodological problem and issue [1]. According to Van de Vijver and Matsumoto [2] the analysis of potential comparability bias is mandatory before concluding that different groups have different scores on the construct under investigation. Comparability bias in surveys on refugees can be an issue, as survey instruments developed in western countries would not represent the concepts in refugees’ cultures or usual western methods such as obtaining ratings would be less familiar to people with no or little experience in taking part in surveys. Therefore, besides the translation issues, refugees’ experiences and background would be associated with biased data and would limit data comparability [3]. Our research therefore focuses on the comparability of measurements in health research between English and Arabic languages in a refugee population.

**Fig. 1:**
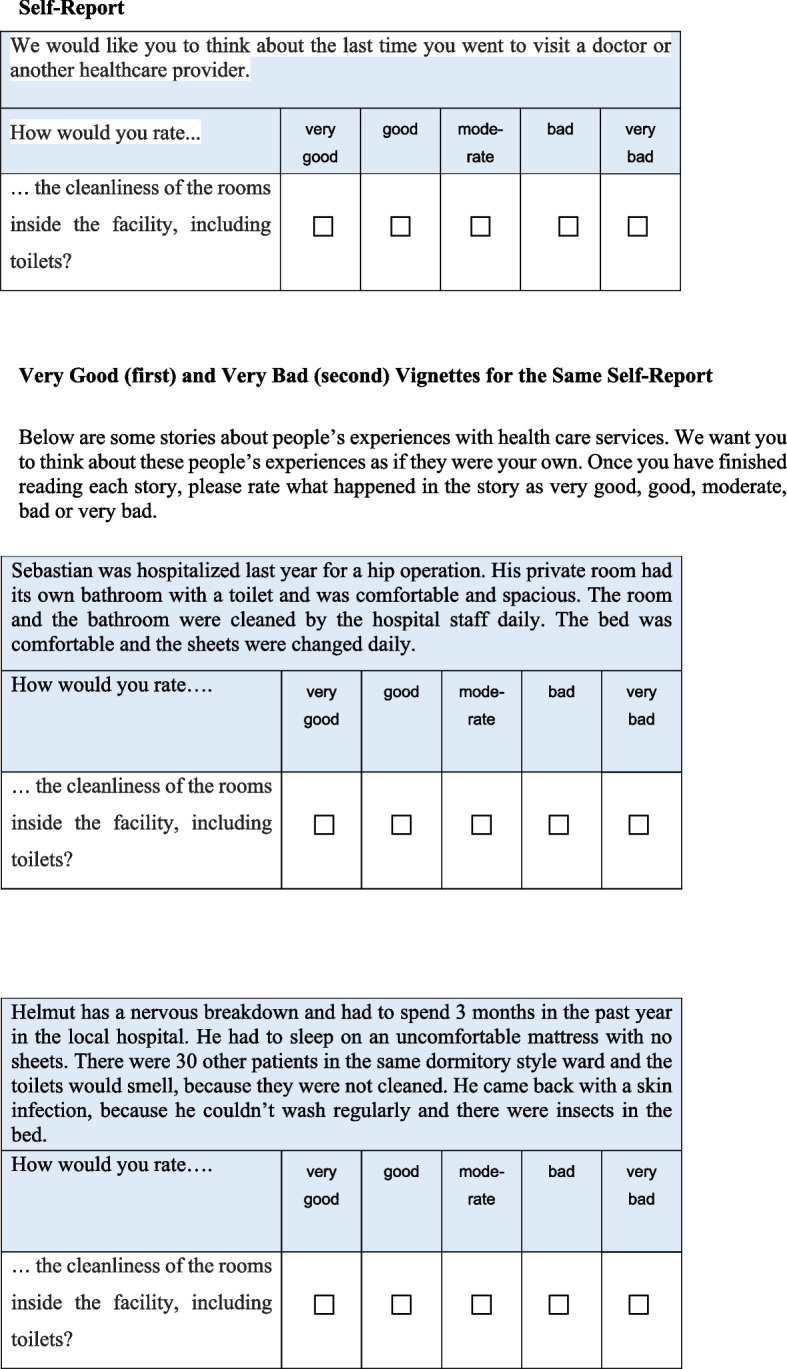
Example for a HSR self-report and anchoring vignettes for the two poles of the rating scale (Very Good, Very Bad)

Information on concepts of interest, such as physical and mental health, well-being, personality, opinions or behaviours have often been collected in surveys by means of multiple indicators (items, questions, manifest variables) that are presented in questionnaires as statements that respondents evaluate with the help of rating scales. Rating scales are graduated response options ordered along a continuum, e.g. ranging from “very bad” to “very good” (example of self-reports and rating scales are provided in Table 1 and Fig. 1). Multiple indicators with rating scales or other response options are used with the promise of measuring unobservable concepts of interest, referred to as latent variables, whereas Latent Variable Modeling (LVM) has been a popular statistical measurement approach [4].

**Table 1:** Indicators of HSR in English questionnaire

Indicator and its label	Question wording
attention (time)	… the amount of time you waited at the doctor’s before being attended to?
respect (resp)	… your experience of being greeted and talked to respectfully?
communication (comm)	…the experience of how clearly health care providers explained things to you? (Language and content easy to understand)
autonomy (aut)	… your experience of being involved in making decisions about your treatment?
confidentiality (conf)	… the way health services ensured you could talk privately to health care providers?
Choice (choice)	… the freedom you had to choose your health care provider?
quality of amenities (clean)	… the cleanliness of the rooms inside the facility, including toilets?

Introduction: The following questions are about your experiences with healthcare services in Germany. If you have not been to a doctor or another medical provider in Germany, please continue with question XX. We are interested in hearing about your experience with healthcare services in Germany. We would like you to think about the last time you went to visit a doctor or another healthcare provider. How would you rate ...

Response options: very good, good, moderate, bad, very bad, cannot say

The development of statistical methods such as Confirmatory Factor Analysis (CFA) within the frame of LVM has enabled the presumption of data comparability to be defined and statistically evaluated, typically by means of multi-group CFA (MGCFA). Statistical evaluation of potential comparability bias has been referred to as measurement invariance analysis. Measurement invariance means that measurement results are not biased by group membership [4], i.e. that individuals with identical individual values on the measured concept or variable provide equivalent manifest responses. Measurement Invariance is rejected, not supported or violated, “if individuals from different groups respond to a test item in a dissimilar manner when they are in the same level on the construct being measured” (p. 524) [5]. Measurement invariance analysis has become increasingly popular in empirical cross-cultural research [6–8], but the results often point to data that is not suitable for the comparisons under investigations, which in turn is associated with data not supporting measurement invariance between different countries and languages [9–13].

Whereas numerous studies report lack of support for measurement invariance in cross-cultural and other group comparisons, little is understood about problems in questionnaires or data collection methods that may lead to comparability bias and rejection of measurement invariance [12]. Van de Vijver [12, 14] differentiates between three kinds of comparability bias: 1) construct bias, meaning that different constructs are measured in different groups; 2) method bias due to sampling, data collection methods and questionnaires used, or 3) item bias, in which items would have different meanings for respondents in different groups. Measurement invariance analysis has been used to identify all these three sources of bias [7, 15].

It is assumed that cross-cultural data comparability can be improved by means of appropriately designed data collection methods and questionnaires [7, 16], which can be expected to have a positive effect on the results of measurement invariance analysis. Previous research has reported on the lack of support for measurement invariance between different data collection modes, i.e. self- and interviewer administration, in the case of grammar or lexical differences in question wording, or for different presentation of response options [7, 17], as well as for the case that respondents understand and use rating scales and response options in different ways [12]. This research provides evidence that data collection methods would be crucial to data comparability. Benitez et al. [12] found rejected measurement invariance associated with different understandings and uses of ratings scales. “Seldom” might therefore refer to very different quantities or the same situation might be evaluated as “very good” by one respondent, but as “good” or even “just satisfactory” by other respondents, depending on respondents’ experiences, habits, motivations, or activated comparability context. In light of the reported difficulty collecting data that are measurement invariant, the question arises, whether there may be ways of ensuring measurement invariance when controlling for the different understandings of rating scales by respondents.

In the present paper we focus on this question and evaluate whether methods that have been used to model the different use of rating scales in other contexts can help to improve the results of measurement invariance analysis and therefore to verify cross-cultural comparability in the data collected. Our first idea is to rely on anchoring vignettes [18, 19], developed within the frame of Item Response Theory (IRT). Anchoring vignettes have been considered as a method of controlling for the varying use or understanding of rating scales by respondents, referred to in the IRT context as Response Category related Differential Item Functioning (RC-DIF). RC-DIF means that individuals with the same value on the latent variable have a different probability of choosing the corresponding answer [20]. RC-DIF can be thought of as the opposite of measurement invariance, as in the presence of RC-DIF measurement invariance might not be supported in the data.

Anchoring vignettes are situation descriptions that correspond to a response category (Fig. 1). “Vignettes represent hypothetical descriptions of fixed levels of a construct… and individuals are asked to evaluate these in the same way that they are asked to evaluate their own experiences…” (p. 175) [21]. In questionnaires, respondents provide both their self-evaluations and evaluations of anchoring vignettes. For the Health System Responsiveness (HSR [21], see Fig. 1, Tables 1 and 2) – the concept we use in the present study – respondents evaluate their own most recent experiences with health care institutions, e.g. timeliness of the last visit to a doctor on a rating scale consisting of “very good”, “good”, “moderate”, “bad”, “very bad”. In addition to this self-report, respondents evaluate vignettes, which are descriptions of fictive situations a person experiences during a visit to the doctor, i.e. waiting for hours in the case of a “very bad” vignette. The RC-DIF is given, if respondents tend to evaluate the vignettes inconsistently with the described level of the concept. This is the case if, for example, the “very bad” vignette situation is evaluated with “bad”, “moderately” “good” or “very good”. Anchoring vignettes are therefore promising in correcting the comparability bias and are becoming increasingly popular. They have been implemented in some large-scale international surveys, i.e. Survey of Health, Ageing and Retirement in Europe (SHARE) [22], Programme for International Student Assessment (PISA) [23], Wisconsin longitudinal Study (WLS) [24], or World Health Survey (WHS) [21]. The HSR instrument for self-reports and anchoring vignettes [21] we use in our study (see Fig. 1 and Tables 1, 2) are taken from the WHS.

**Table 2:** Survey question and anchoring vignettes of the HSR indicator “quality of basic amenities”

**Introduction**
Below are some stories about people’s experiences with health care services. I want you to think about these people’s experiences as if they were your own. Once you have finished reading each story, please rate what happened in the story as very good, good, moderate, bad or very bad
**Rating:**
How would you rate the cleanliness of the rooms inside the facility, including toilets?
Very good, Good, Moderate, Bad, Very bad
**Very Good Vignette**
Sebastian was hospitalized last year for a hip operation. His private room had its own bathroom with a toilet and was comfortable and spacious. The room and the bathroom were cleaned by the hospital staff daily. The bed was comfortable and the sheets were changed daily
**Good Vignette**
Anne had her own room in the hospital and shared a bathroom with two others. The room and bathroom were cleaned frequently and had fresh air
**Moderate Vignette**
Martina shared a hospital room with four other persons. There was a toilet for her ward located along the outside corridor, which occasionally wasn’t clean. The room was only occasionally dusty
**Bad Vignette**
Dirk shared his small hospital room with five other patients with no partitions between beds. The five patients shared a toilet, which was cleaned every second day and sometimes smelt
**Very Bad Vignette**
Helmut has a nervous breakdown and had to spend 3 months in the past year in the local hospital. He had to sleep on an uncomfortable mattress with no sheets. There were 30 other patients in the same dormitory style ward and the toilets would smell, because they were not cleaned. He came back with a skin infection, because he couldn’t wash regularly and there were insects in the bed

As anchoring vignettes can help to controll or adjust the data for RC-DIF, their use can also influence the results of measurement invariance analysis by means of MGCFA and therefore help to establish cross-cultural comparability in the existing data. He et al. [25] and Marksteiner et al. [23] demonstrated a more satisfactory model fit of measurement invariance analyses when data was rescaled using anchoring vignettes in PISA. However, the results of these studies are mixed and more research is needed, especially when it comes to health-related topics and refugee populations. The implementation of anchoring vignettes also requires that additional information is asked for in questionnaires. This would be burdensome, particularly if a full set of vignettes (e.g., five in the case of five category rating scales) is used for each indicator. For the seven indicators of the HSR short scale, 35 vignettes should be additionally included in the questionnaire. This limits the use of vignettes in the survey practice and makes alternatives relevant. Hox et al. [26] use demographic information as covariate variables in the MGCFA measurement invariance analysis when comparing different modes of data collection. In a similar way, we evaluate whether the use of demographic information in MGCFA models can help to reduce comparability bias and improve cross-cultural comparability as an alternative to anchoring vignettes.

Our research addresses the research question on how to ensure cross-cultural comparability should this be found to be violated in the data. We evaluate whether the use of anchoring vignettes or solely socio-demographic information enables more satisfactory results of measurement invariance analysis to be obtained. We use data collected in Germany on HSR when evaluating cross-cultural comparability between refugees responding in English or in Arabic. Extending on previous research [23, 25], we address a health research topic among refugee populations. In doing so, we replicate the studies by He et al. [25] and Marksteiner et al. [23]. We additionally consider MGCFA covariate models incorporating information on RC-DIF predicted from vignettes’ ratings. Further, we consider demographic variables without information on RC-DIF from vignettes’ ratings.

Our paper is structured as follows. We firstly provide specifications of measurement invariance models, describe parametric and non-parametric approaches to the use of anchoring vignettes and present our research questions. Secondly, we describe the study, data and materials as well as data analysis methods. Third, we provide the results. Finally, we discuss the results and draw conclusions.

### Measurement invariance

Measurement invariance analysis provides information on whether between-group comparisons of latent variables or summarized scores deliver valid results, as certain levels of measurement invariance point to bias free statistical comparisons [4, 13, 27]. Measurement invariance analysis is typically conducted by a sequence of steps of MGCFA.

In MGCFA, a measurement model (that is a CFA model) is evaluated for observed scores *Y* on an indicator of individual *i* within group* j*:

1 $$Y_{ij}=\tau_j+\Lambda_j\eta_{ij}+e_{ij},$$

where *τ*_*j*_ represent intercepts and *Λ*_*j*_ represent factor loadings for the group *j*, and *η*_*ij*_* and e*_*ij*_ represent common scores and residuals for the individual *i* in group *j*, respectively.

The following increasing degrees (or levels) of measurement invariance are relevant to the survey context [26, 27],^1^ with each subsequent one including the preceding [4, 27]:

- Configural invariance is defined in Eq. (1) and holds when the number of factors (latent variables) and indicators per factor are comparable across groups. If the configural invariance holds (that is, not rejected by the data), however, statistical comparisons of latent variables or simple sum scores are not sensible among groups.
- Metric or weak invariance holds, if *Λ*_*j*_ = *Λ* for all groups, that is, if loadings that reflect the strength of associations between the manifest and latent variables are comparable among groups. Metric invariance should be given to exclude comparability bias as an alternative explanation when comparing correlations among groups. To evaluate metric invariance, equality constraints on factor loadings among the groups are introduced into the configural model. Equality of factor loadings and therefore the presence of metric invariance is proven if the introduction of the restriction does not significantly decrease model fit.
- Finally, scalar or strong invariance among groups holds, if *τ*_*j*_ = *τ*. Scalar invariance is evaluated by restricting the intercepts of the manifest variables to make them equal among groups. Again, this restriction should not significantly decrease model fit. Satisfying scalar invariance allows for exclusion of measurement bias as alternative explanation and therefore valid comparisons of both latent mean scores and means of summarized scores.

This description of different degrees of measurement invariance shows, therefore, that weak and strong measurement invariance are prerequisites of the bias-free cross-group comparisons, as their violation means that results are confounded with the group comparability bias in the measurement.

With respect to measurement invariance in cross-cultural studies, researchers often fail to support strong or even weak invariance in their data [14], as shown for the Trends in International Mathematics and Science Study (TIMMS) [13], for different concepts of the European Social Survey (ESS) [9, 11], or for some of the concepts in the International Social Survey Program (ISSP) [28]. Dong and Dumas [29] report in a meta-analysis that scalar invariance between ethnic groups was not supported for any of the personality inventories considered. One line of research tried to develop less restrictive data analysis methods [30, 31], while the other line of research has been targeting the question as to which circumstances of data collection or cognitive respondents’ problems are associated with the rejection of statistical measurement invariance [7, 12, 16].

Differences in response behaviour can be systematically described using the theory of the cognitive response process [32] that comprises four separate steps when answering a survey item: The comprehension of a survey question, information retrieval, judgement, and finally, response according to the given response options. When using rating scales, cross-cultural differences in response behaviour during the last step of the cognitive response process would manifest in response styles or response sets, such as acquiescence [33], or middle and extreme response tendencies [34]. Previous research identified cross-cultural differences in response tendencies depending on education, acculturation, or Hofstede’s dimensions of individualism, power distance or masculinity [35]. Response styles and response sets may bias the data and limit their comparability, with the manifestations in rejecting measurement invariance in the corresponding statistical models.

Knowing such sources sheds more light on the sensitivity of the measurement invariance modeling and practical significance of its results [14]. Rejection of metric invariance, for instance, would imply that extreme response style is present in the data [36–38]. Rejecting scalar invariance would point to the presence of additive systematic measurement error, such as acquiescence [38]. Benitez et al. [12] found rejection of both, metric and scalar measurement invariance explainable by RC-DIF. In addition, research on rating scales has shown that use of different numbers of categories or different category labelling lead to the rejection of metric and scalar invariance [17, 39].

### Modelling and controlling RC-DIF by means of anchoring vignettes

Data can be adjusted for RC-DIF using anchoring vignettes when a parametric or a non-parametric approach is implemented [18, 40]. In case of a non-parametric approach, vignette assessments (*z*) are used to rescale the self-assessments (*y*). *J* is the notation for the number of vignettes (*j* = 1, …., J). The rescaling produces a new variable *C*. (Eq. 2) [18, 40]:

2 $$C= \left\{\begin{array}{c}1 \quad if \quad y< {z}_{1}\\ 2 \quad if \quad y={z}_{1}\\ 3 \quad if \quad {z}_{1}<y< {z}_{2}\\ . \\ . \\ . \\ 2J+1 \quad if \quad y< {z}_{j}\\ \end{array}\right.$$

In the case of a correct ordering of vignettes, *C* is a scalar variable. In the case of misallocation, *C* can obtain different possible values and represents an interval. For example, in the case of two vignettes *z*_1_ and *z*_2_ and *y* = *z*_1_ = *z*_2_ *C* obtains values 2, 3, and 4 [40]. The use of rescaled data for measurement invariance analysis is referred to in the following as non-parametric data adjustment.

The parametric approach, on the other hand, uses a hierarchical ordered regression model (abbreviated CHOPIT) to predict respondents’ self-assessment (*s*) by their evaluation of vignettes (*v*) [18, 40]. In this approach, a respondent (denoted by *i* = 1, …, *N*) has an unobserved level ($${Y}_{i,s}^{*})$$ of his/her self-assessments (*s* = 1, …., S), given the actual observed level of self-evaluation ($${\mu }_{i}$$), as shown in Eq. 3.

3 $$Y_{i,s}^\ast\sim N\left(\mu_i,\sigma_s^2\right).$$

The actual level ${\mu }_{i}$ is a linear function of observed covariates *X*_*i*_ (e.g. gender, age, education), see Eq. 4.

4 $${\mu }_{i}={X}_{i}\beta +{\eta }_{i},$$

where $\beta$ is the parameter associated with the impact of covariates and $\eta$ the normal random effect.

The reported survey response ${y}_{i,s}$ is also dependent on the chosen response category *k* (*k* = 1,…, K_s_) as follows:

5 $${y}_{i}=k, \quad if \quad {\tau }_{i,s}^{k-1}\le {Y}_{i,s}^{*}<{\tau }_{i,s}^{k},$$

where ${\tau }_{i,s}$ is a vector of ordered thresholds (ranging from -∞ to + ∞). The thresholds are defined as follows (Eq. 5):

6 $${\tau }_{is}^{1}= {\gamma }_{s}^{1}{V}_{i}$$

${\tau }_{is}^{k}= {\tau }_{is}^{k-1}+{e}^{{\gamma }_{s}^{k}{V}_{i}}$, where *V*_*i*_ is a vector of covariates and ${\gamma }_{s}^{k}$ a vector of unknown threshold parameters.

For the vignettes, there is also a predicted value for each respondent from the observed vignette value *θ*_*j*_, while respondents are denoted with *l*:

7 $$Z_{li,s}^\ast\sim N\left(\theta_j,\sigma_{sj}^2\right).$$

The observed vignette values (*z*) depend on response categories as follows:

8 $${z}_{lsj}=k, \quad if \quad {\tau }_{ls}^{k-1}\le {Z}_{ls}^{*}<{\tau }_{ls}^{k},$$

Correspondingly, the values of vignette thresholds are predicted as follows:

9 $${\tau }_{l1}^{1}= {\gamma }_{s}^{1}{V}_{l}$$

$${\tau }_{l1}^{k}= {\tau }_{ls}^{k-1}+{e}^{{\gamma }_{s}^{k}{V}_{l}}.$$

In both self-reports and vignette components, the thresholds vary on the same covariate variable components (*X*_*i*_ vs *V*_*l*_). The CHOPIT model estimates in parallel the self-component (mean location of self-assessments), the vignette component (mean location of the vignettes) and thresholds for the self-assessments given vignettes’ evaluations. The use of estimates for vignette components in other models is referred to in the following as parametric adjustment by means of anchoring vignettes.

One line of research on anchoring vignettes addresses the possibility of evaluating the general assumptions of their use, namely vignette consistency and vignette equivalence [22, 41]. Vignette consistency assumes that response behaviour is the same in the case of vignette evaluations and self-assessments, while vignette equivalence means that the same latent dimension explains the responses to all vignettes. The equivalence needs to hold not just within the vignette set, but – in light of response consistency – also between vignettes and self-report questions [42–44]. Research has particularly evaluated vignette consistency when using correlations with third variables, and the use of objective measures for these variables has been suggested as the best solution [18, 22, 41]. The results from fulfilment of these general assumptions have been mixed, however [44].

Research has also been conducted on the usability of vignettes to actually improve the comparability of data (i.e. adjust for the RC-DIF). One relevant finding is that adjustments with vignettes were associated with a higher criterion validity. King et al. [18] showed this for visual ability, van Soest et al. [41] for drinking behaviour and Mottus et al. [45] for a personality measure. However, He et al. [25] obtained mixed results with respect to validity coefficients. Marksteiner et al. [23] found a higher internal consistency of rescaled data when using the non-parametric rescaling for non-cognitive skills of students in PISA.

The vignettes’ effect on RC-DIF and adjustment of data for comparability – the specific aim of the vignette approach – has been mainly evaluated by comparing adjusted and non-adjusted results (both, parametric modelling and non-parametric rescaling), obtaining more plausible conclusions when using anchoring vignettes [19, 45]. However, such a comparison does not allow for a statistical test and therefore does not provide strong evidence that anchoring vignettes affect (cross-cultural) comparability. By way of contrast, measurement invariance analysis (as described in the previous section) allows the suitability of data for statistical comparisons to be tested directly. The research that applies MGCFA models on rescaled data (with non-parametric rescaling, Eq. 2) is available for PISA. He et al. [25] found a slightly reduced difference in the model fit when evaluating metric invariance. The authors also found the inconsistent use of anchoring vignettes to be correlated with low socio-economic status and low cognitive skills, which point to the relevance of these factors for comparability bias. Marksteiner et al. [23] also used PISA data on non-cognitive skills and found a higher level of measurement invariance for rescaled data (non-parametric rescaling, Eq. 2) for some contents, but not for others. The authors conclude that the effect of rescaling on the basis of anchoring vignettes on the results of measurement invariance may be dependent on the topic. They also suggest further research when using parametric approach.

### Research questions

So far, we can state that on the one hand, research has often found a comparability bias in cross-cultural large-scale surveys such that strong or even weak measurement invariance are rejected in the data. On the other hand, anchoring vignettes have been used as an approach of control of RC-DIF and it can be expected that information on RC-DIF from anchoring vignettes is utilisable for measurement invariance analysis. Previous research [23, 25], supported this assumption for PISA data in educational research. We extend previous research by addressing both, a health topic and a refugee population, further implementing the parametric modelling. As outlined earlier, the parametric approach makes particular use of socio-demographic and other respondents’ background variables (covariates, see Eqs. 6, 9). The administration of anchoring vignettes may depend on cognitive skills [25] and response styles, and the latter were found to be dependent on socio-demographic variables [34]. In other contexts, consideration of socio-demographic variables helped in supporting assumptions of measurement invariance, i.e. when evaluating mode effects in non-experimental data [26]. Therefore, when applying the parametric approach, the potential effect on measurement invariance can be due to both, socio-demographic information and anchoring vignettes, which should be separated from each other. This also has practical consequences, if comparable results with respect to measurement invariance are obtained incorporating socio-demographic information. If so, socio-demographic information can be used to control for RC-DIF thereby avoiding the workload associated with anchoring vignettes.

With this in mind, we address the research question on the extent to which anchoring vignettes can be used to accomplish cross-cultural comparability of data. More concretely, we respond to the following research questions:

1. How does information on RC-DIF obtained from anchoring vignettes alter the results of measurement invariance analysis?
2. Does implementing non-parametric rescaling and incorporating CHOPIT-predictions into the analysis of measurement invariance provide similar results with respect to configural, metric and scalar invariance?
3. Are these results comparable to those that consider socio-demographic covariates only?

## Methods

### Data

This analysis uses data from a population-based, cross-sectional survey among refugees living in collective accommodation centres in the German state of Baden-Württemberg, conducted as part of the RESPOND project *(‘Improving regional health system responses to the challenges of migration through tailored interventions for asylum-seekers and refugees’* – RESPOND) from February to June 2018. The development of the questionnaire, the sampling and data collection approach have been described in detail elsewhere [46, 47]. The pen and paper questionnaire comprised established instruments covering health status, utilization of health services, HSR (incl. corresponding anchoring vignettes), as well as several socio-demographic characteristics. It was developed in German and English and translated into the refugee languages (among others into Arabic, which is relevant to this paper) using a team approach [48]. The questionnaire was subsequently assessed in the form of a cognitive pretest and refined accordingly [49].

Sampling of participants was conducted on the basis of residential units which included initial reception and regional accommodation centres as no population-based registry of all asylum seekers in the state was available for research purposes. A two-stage sampling design was employed for initial reception centres: First, six of nine centres were purposely selected based on their size, geographical location and administrative responsibility. Second, 25% of rooms (depending on their occupation status) were randomly selected. For regional accommodation facilities, a record of all 1938 facilities in the state was compiled and a random sample of 65 facilities drawn, balancing the number of refugees in each accommodation facility. All individuals living in the selected rooms (reception facilities) or facilities (regional accommodation centres) who could speak one of the study languages and were 18 years or older were invited to participate. The probabilistic clustered sample design is chosen to allow representation of the refugees in the federal state of Baden-Wuerttemberg in Germany, whereas it was also shown that the composition of refugees in the sample was comparable to that in the population [46, 47], which means that there was not a considerable sample bias for the researched refugee groups.

Data was collected by trained, multilingual staff visiting each selected accommodation facility on two consecutive days. Eligible individuals were approached in person by the research staff, who explained the purpose of the study with the aid of pre-recorded audio-messages where there were language barriers. The staff distributed information leaflets, the questionnaires as well as non-monetary, unconditional incentives. Participants could either return the questionnaire to the research staff in person or by post in a stamped envelope. All methods were carried out in accordance with relevant guidelines and regulations, such as ethical standards and the data protection regulations of the European Union (GDPR). All persons were provided with detailed information on the purpose and content of the study, voluntary participation, data collection purpose, data handling and participants’ rights. Informed consent was obtained from all study participants.

Out of 1429 eligible individuals, 1201 were invited to participate in the study. A total of 560 participants completed the survey (reception centres: 149; accommodation centres: 411), with a total response rate of 39.2%. This response rate is satisfactory due to decreased participation rates in surveys, while response rates of 30% or lower are rather usual [50, 51]. Since anchoring vignettes for HSR are implemented in English and Arabic only, the analyses are necessarily restricted to these two groups. As sample bias was not present in the entire sample, it does not arise in the selected groups either. Of those respondents who used English to participate in the study (*n* = 183), 27% were from Gambia, 43% from Nigeria, 6% from Sri Lanka and 16% had other countries of origin that were not specified further in the questionnaire. Of the Arabic speaking persons (*n* = 121), 56% were from Syria, 26% from Iraq and 14% of other origin. Table 3 provides further information on the socio-demographic characteristics of our sample (*N* = 304).

**Table 3:** Summary of Sample Characteristics

	share in %	n
English speaking	60	183
Arabic speaking	40	121
Female	23	69
Age 26–30	19	59
Age 31–35	15	45
Age 36–40	9	28
Age 41 +	11	33
Insurance electronic Card yes	38	114
Insurance electronic Card – missing data	6	19
Education no completed school^a^ / do not know	25	75
Education mandatory school	19	59
Education high school	35	105

*N* = 304 (100%) total English and Arabic speaking persons, *N* = 245 who administered HSR and vignettes

^a^Permission from third persons was not relevant, as all respondents are adults (older than 18 years) and participated with their own explicit consent

### Material

HSR is defined as “aspects of the way individuals are treated and the environment in which they are treated during health system interactions” (p.138) [21]. The inventory aims to measure the latent concept of the non-technical quality of care received during healthcare interactions, including respectful and confidential treatment by health care personnel, clarity of communication and information, timeliness of treatment and the quality of basic amenities. HSR was first implemented in the WHO Multi-Country Survey Study and subsequently embedded in the World Health Survey (WHS), collecting data in over 70 countries. It is currently part of the WHO Study on global ageing and adult health (SAGE). However, it has not previously been used specifically in refugee populations [52] and no analysis of measurement invariance for HSR has previously been available. HSR utilizes a five-category, fully verbalized rating scale ranging from “very bad “ to “very good “ (Table 1). In addition, anchoring vignettes for the HSR are used in WHS, making HSR particularly relevant for the aim of our study (Table 2).

We used the short-form version of HSR included in the WHS, restricting our questions to ambulatory care only (see Table 1 for the question wording). The HSR instrument as implemented in the WHS demonstrated moderate test–retest reliability (kappa values of 0.40–0.49 across domains) and border internal consistence (Cronbach’s alpha of 0.65) [21].

Prior to data collection, the translated version of the HSR instrument was included in a cognitive pretest [49]. Using probing and think-aloud techniques, these pretests evaluated the intelligibility of the items and assessed potential unintended misunderstandings with nine refugees in five languages, including English and Arabic. This pretest resulted in the simplification of the question format and clarifications of particular terms used. The reliability of the improved HSR as a latent dimension was sufficiently high in the whole asylum seekers sample (factor analysis based reliability [53] *ro* = 0.87; all loadings were higher than 0.50).

Respondents evaluated vignettes in addition to self-assessment on HSR (see Table 2 for an example of vignettes). Using vignettes for each of seven indicators and each response category resulted in 35 vignettes, which means that an additional 35 questions had to be included in the questionnaire. To reduce the workload of respondents and due to the limited number of questions that could be included in the survey, we used five different sets of vignettes in each language, with each set being randomly assigned to a respondent group. The first set of 21 vignettes contained the top, the middle and the bottom vignettes for each of the HSR indicators. The other four sets included five sets of vignettes for each response category for two or one of the indicators of HSR (set two attention and respect, set three communication and quality of amenities; set four confidentiality and choice; set five autonomy).

### Data analysis

For the HSR, we conducted MGCFA analyses with the software Mplus 8.7. To compare loadings and intercepts, the factor means were set to 0 and variances to 1 [36, 54]. In the case of ordinal data with five to seven categories and small samples, Robust Maximum Likelihood estimator on the basis of Pearson correlation (MLR) [55] provides more stable and valid results than use of estimators for categorical data, such as weighted least squares WLSMV estimator on the basis of polychoric correlations [56]. We therefore mainly relied on MLR to account for ordinality and non-normality of data. To validate the results, we also conducted analyses for ordinal (meaning ordered categorical data) when using WLSMV estimator [55].

The model fit of MGCFAs was evaluated using the chi-square test (CMIN), the Root-Mean-Square Error of Approximation (RMSEA), and the Comparative Fit Index (CFI) [57]. The CFI should be 0.95 or higher, while the RMSEA of 0.08 or less indicates an acceptable fit [58]. A significant change of CMIN [4] or a change of ΔCFI ≥ 0.005 and ΔRMSEA ≥ 0.010 indicate significant differences in model fit if the samples are small (n < 300) and unequal [59], thus demonstrating a lack of measurement invariance. To compare the different unnested models with different covariate variables and those with different sample sizes, sample size adjusted Bayesian Information Criterion (BIC) was used, where lower values indicate a better model fit and a change of BIC ≥ 6 indicates a significant change [60]. BIC is also used as the main statistic to compare models for ordered categorical data analysis (WLSMV estimator) by means of mixture modelling, since other model fit statistics are not available for them [55].

#### Measurement invariance analysis with data rescaling (non-parametric approach)

For the non-parametric approach, we used the rescaling procedure for each of the HSR indicators as introduced in Eq. 2. Because the non-parametric approach requires each respondent to evaluate vignettes (while a selection of vignettes can be used, i.e. top or bottom or top, middle, bottom [23]), we calculated means for top, middle and bottom vignettes from all respondents’ groups (see information on different groups using different vignette sets above). The analysis to rescale the self-reports on HSR indicators was conducted using R [61] (see Eq. 2; the software source is included in Suppl. Mat. 1). A similar procedure, also using two or three vignettes (top, middle, bottom) was implemented in He et al. [25] and Marksteiner et al. [23]. The anchor package of R accounts for the inappropriate ordering of vignettes and predicts C variable including the lowest (Cs) and highest possible (Ce) ratings for each of the HSR items. Similar to He et al. [25] and Marksteiner et al. [23], we considered both predictions. The rescaled variables were subsequently used in MGCFA analyses to evaluate measurement invariance.

#### Measurement invariance analysis with covariates

In addition, we evaluated the RC-DIF by means of the parametric approach (Eqs. 3, 4, 5, 6, 7, 8 and 9), using glamm function of Stata [24, 62] (Suppl. Mat. 2). Data from the groups who used vignette sets 2 to 5 was included. In the CHOPIT analysis, the covariates were language, gender, age, and having an electronic health insurance card. We considered these variables to be relevant to health care experiences. With respect to other variables, such as economic, occupational status or living conditions, the respondents were deemed to be too similar due to their status as asylum seekers and their current stay in refugee centres. Also, our small sample size prohibited the use of too many predictors. As the education variable had a reasonable number of missing values (*n* = 21 English and *n* = 15 Arabic), we excluded it from the CHOPIT analysis to avoid a substantial decrease of sample size. To be able to use the vignette data in the MGCFA, we saved the predicted threshold parameters (Eq. 9, see glamm code in Suppl. Mat. 2). The results of the CHOPIT analysis for an example indicator of the HSR can be found in Suppl. Mat. 3, as these are out of our focus and the procedure was merely used as an auxiliary step to predict thresholds for subsequent use in the MGCFA.

There are no solutions in the literature for the implementation of corrections when relying on the parametric approach within anchoring vignette research. However, within LVM in Structural Equation Modeling (SEM), one method of controlling for sample heterogeneity has been to include covariate variables in the analysis [63, 64].Therefore, in the MGCFA model of HSR we included predicted threshold parameters as covariates which were regressed on observed indicators. So, in our models, the variation in observed indicators is explained by both the latent variable and by vector of covariates. Such models have been referred to as covariate models within CFA [63]. To define our model we extend the Eq. (1) as follows:

10 $${Y}_{ij} = {\tau }_{j} + {\Lambda }_{j}{\eta }_{ij} +{\Gamma }_{j}{x}_{ij} + {e}_{ij}$$

where *x* are covariates and *Γ*_*j*_ are the regression weights. The covariates are either the predicted thresholds from the CHOPIT analysis or socio-demographic variables. We did not postulate a second latent variable to explain covariates, since the socio-demographic variables are not expected to build a latent variable. Similarly, the response thresholds predicted from the vignettes can be explained by a latent variable if vignette equivalence holds. To overcome this assumption, we considered predicted indicator thresholds as manifest covariates (Eq. 10). Overall, CHOPIT predicted four thresholds for each of seven indicators of HSR, which means having a vector of 28 covariates in Eq. 10, which challenges the model complexity and small sample sizes we had. We therefore included predicted thresholds with a significant path on at least one indicator of the HSR. The resulting covariate model is shown in the main text in Fig. 4 and the software code is provided in Suppl. Mat. 4. The covariate MGCFA model with socio-demographic variables is shown in Fig. 5; see additionally Suppl. Mat. 4 for the Mplus code.

## Results

### Vignettes accuracy

**Fig. 2:**
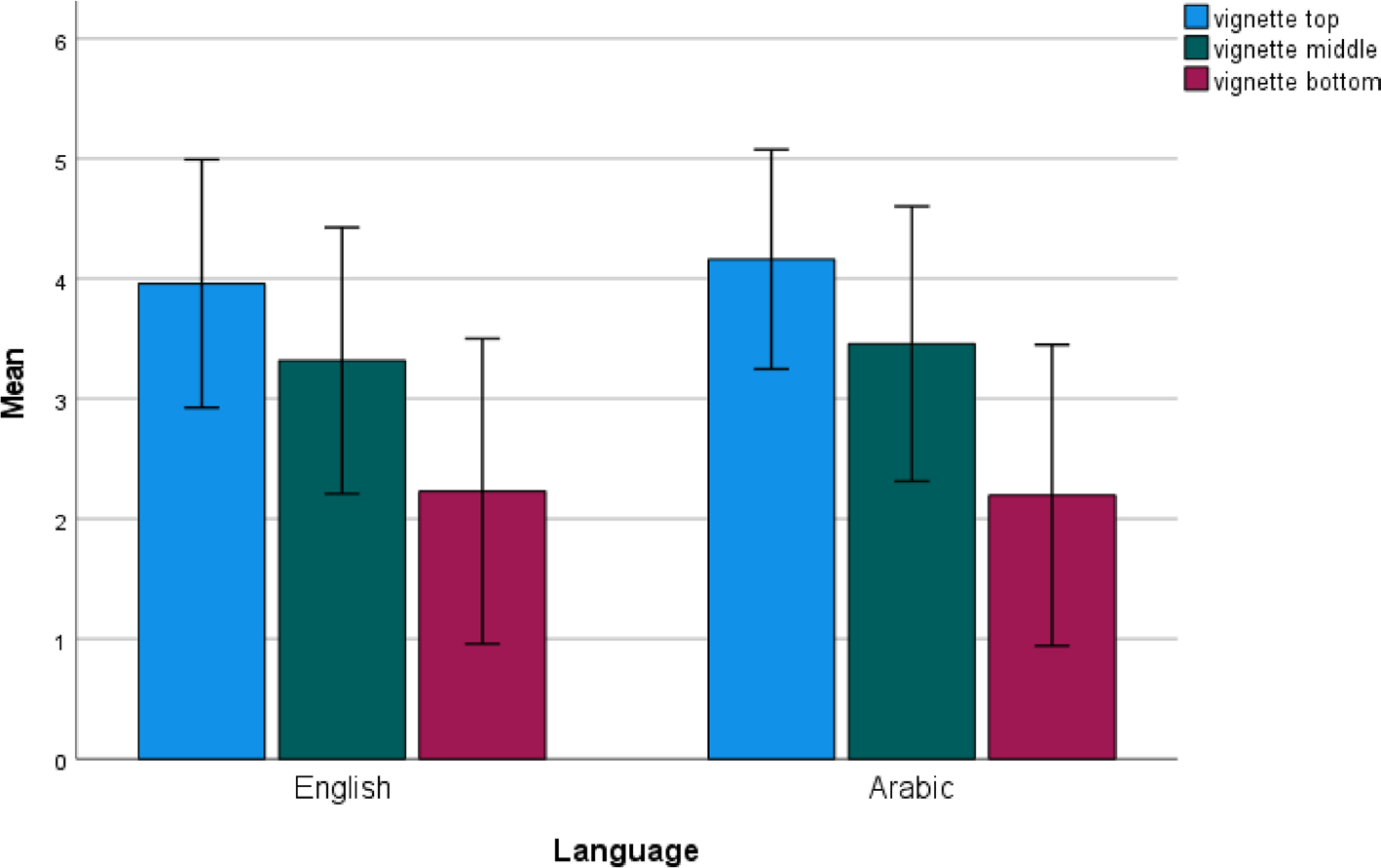
Evaluation of the top, middle and bottom vignettes by language

The proportion of accurately ordered vignettes was 68% in the English speaking group and 76% in the Arabic speaking group. It can be seen from Fig. 2 that vignettes were evaluated similarly by the two language groups, and according to a MANCOVA (Multivariate Analysis of Covariance) there were no significant differences in the languages between the mean evaluation of bottom, moderate and top vignettes (Pillai's Trace *(PT)* = 0.01; *F*_(3,228)_ = 1.04, *p* > 0.10, *η*^*2*^ = 0.01). There were also no significant differences in the evaluation of vignettes between men and women (*PT* = 0.02; *F*_(3,217)_ = 1.26, *p* > 0.10, *η*^*2*^ = 0.02), or between different age groups (*PT* = 0.07; *F*_(9,636)_ = 1.57, *p* > 0.10, *η*^*2*^ = 0.02). However, respondents with higher education ranked the top (*M* = 4.23; *SD* = 0.82) and bottom (*M* = 1.97, *SD* = 1.1) vignettes more consistently (*PT* = 0.10; *F*_*(6, 424)*_ = 3.61, *p* < 0.01, *η*^*2*^ = 0.02). Finally, respondents without electronic health insurance cards evaluated the “very good” (top) vignette closer to its rank 5 (*M* = 4.16, *SD* = 0.91) than other respondents (univariate effect F_(1,230)_ = 3.84, *p* = 0.05, *η*^*2*^ = 0.02).

### Measurement invariance

#### Initial model of HSR

**Fig. 3:**
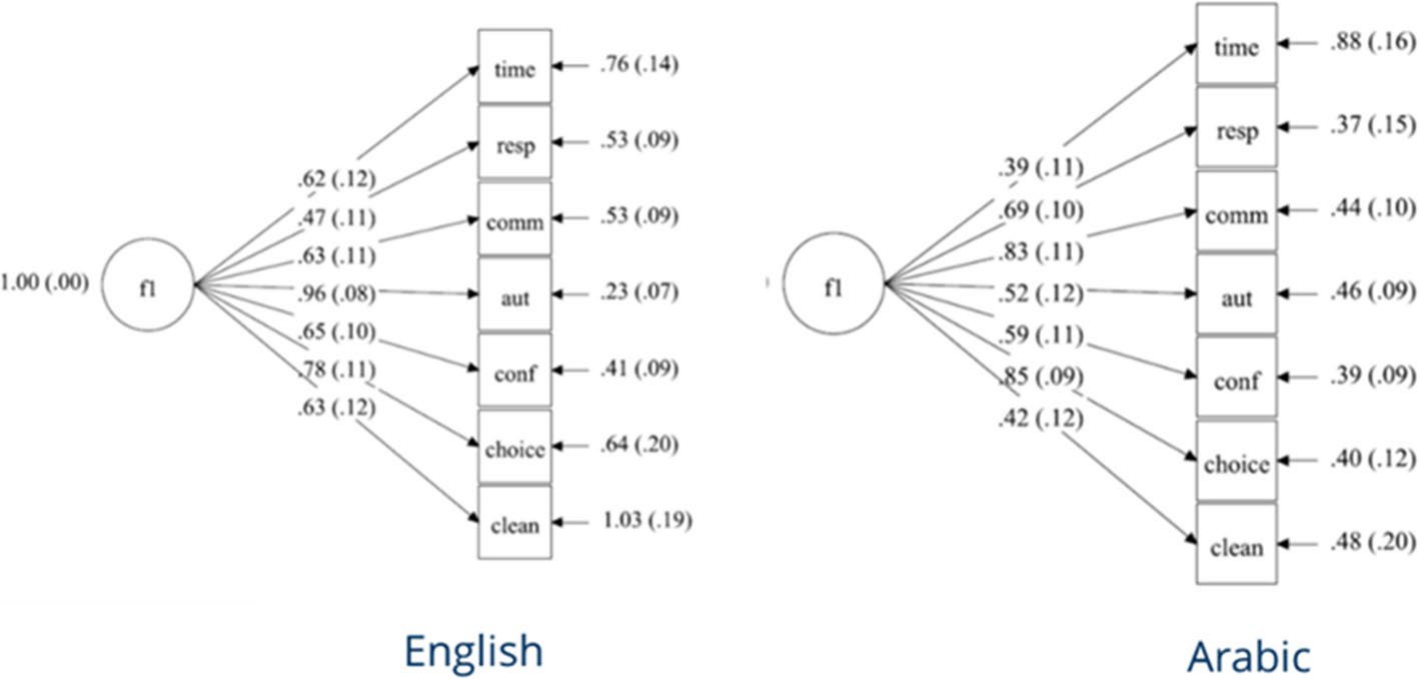
Initial HSR MGCFA configural model in english and arabic languages Note. f1 factor HSR

Figure 3 shows the MGCFA model and Tables 4 and 5 provide an overview of estimated parameters for the configural model of HSR, while Table 6 provides goodness of fit statistics. The configural invariance was not supported, because the configural model was associated with a pure model fit due to the significant CMIN, the RMSEA reasonably above and the CFI far below the benchmark (Table 6). In other settings and according to the steps of measurement invariance analysis, the procedure ends by rejecting a level of measurement invariance. To be able to compare the results for not adjusted and adjusted models, however, and to identify which levels of measurement invariance would be affected by the adjustment, it was important to obtain the results for the cross-cultural comparability of loadings and intercepts of the HSR. For this, we inspected misspecifications looking at the Modification Indexes (MIs, which describe the decrease of *CMIN* if a modification that is a deviation from the initial model is introduced; procedure proposed e.g. by Byrne [54]). According to the high sizes of MIs, we successively introduced correlated errors, first between the items “attention” and “respect” and second between “respect” and “communication”. To allow for the comparability of the modified configural models between the two language groups, the introduced modifications (error covariances) were held equal between them (see Suppl. Mat. 4 for the model specification). The modifications led to an acceptable fit of the multi-group model due to *CFI* and *RMSEA*. This result supports the violation of the one-dimensional structure for the HSR, but shows that this was uniform in both languages. It should be kept in mind, that ad-hoc modifications restrict the comparability of the results to other samples and populations, whereas new analyses in other samples are needed to verify the modifications. The aim of this analysis was not to accomplish the next level despite the violation of the previous level, but to obtain information on the potential comparability bias on all levels of measurement invariance.

**Table 4:** Non-standardized loadings of configural models of HSR and covariate models

	*Initial*		*Non-Parametric Rescaling*		*Covariate vignette thresholds*		*Covariate SD*	
HSR Indicators	*E*	*A*	*E*	*A*	*E*	*A*	*E*	*A*
attention	0.55	0.34	1.95	1.53	0.57	0.46	0.51	0.31
respect	0.40	0.62	2.09	2.22	0.40	0.63	**0.31**	**0.65**
communication	0.60	0.80	1.60	1.53	0.60	0.72	0.54	0.82
autonomy	**0.99**	**0.55**	1.75	1.35	**0.94**	**0.40**	**0.93**	**0.53**
confidentiality	0.66	0.62	2.11	2.00	0.62	0.48	0.60	0.64
choice	0.79	0.89	1.84	1.45	0.81	0.75	0.77	0.89
amenities	0.60	0.41	2.06	2.24	0.51	0.37	0.50	0.37

Bold non-invariant parameters

*SD* Socio-demographic variables, *E* English, *A* Arabic

**Table 5:** Non-standardized intercepts of configural models of HSR and covariate models

	*Initial*		*Non-Parametric Rescaling*		*Covariate vignette thresholds*		*Covariate SD*	
HSR Indicators	*E*	*A*	*E*	*A*	*E*	*A*	*E*	*A*
attention	**3.64**	**3.46**	2.80	2.23	2.81	4.00	3.68	3.34
respect	4.22	4.28	3.71	3.32	2.62	2.78	4.29	4.22
communication	**4.00**	**3.83**	3.19	2.46	**4.32**	**2.96**	3.87	3.65
autonomy	3.78	3.79	2.94	2.45	3.84	3.89	3.63	3.68
confidentiality	**3.97**	**4.21**	3.07	3.03	2.70	3.07	3.81	4.12
choice	3.76	3.76	2.92	2.37	1.04	2.61	3.69	3.97
amenities	**3.95**	**4.49**	3.56	3.63	2.35	5.45	4.16	4.49

Bold non-invariant parameters

*SD* Socio-demographic variables, *E* English, *A* Arabic

**Table 6:** Measurement invariance analysis for HSR without and with adjustment

model	*χ*^*2*^*(df)*	*Δχ*^*2*^*(df)*	*RMSEA*	*ΔRMSEA*	*CFI*	*ΔCFI*	*BIC*
	Initial						
configural	65.47 *** (28)	-	.106	-	.887	-	3599.87
configural modified	41.27* (26)	-	.071	-	.954		3574.58
metric	62.02** (33)	20.25** (8)	.086	.015	.912	.042	3582.79
scalar	90.93*** (40)	29.38*** (8)	.104	.018	.846	.066	3599.09
	n (English) = 145; n (Arabic) = 91						
	Adjusted Non Parametric Highest Ratings						
configural	53.98** (28)	-	.097	-	.850	-	3458.44
metric	64.42** (35)	10.80 (24)	.092	.005	.830	.020	3480.28
scalar	78.21** (42)	15.75** (6)	.093	-.001	.791	.039	3478.99
	n (English) = 117; n (Arabic) = 81						
	Adjusted Non Parametric Lowest Ratings						
configural	43.67* (28)	-	.075	-	.954	-	4872.06
metric	51.90* (35)	6.77 (8)	.070	-.005	.951	.003	4865.25
scalar	65.59* (42)	16.37** (6)	.075	.005	.931	.020	4865.85
	n (English) = 117; n (Arabic) = 81						
	Adjusted Covariate Model CHOPIT Vignette Thresholds						
configural	80.22 (64)	-	0.052	-	.946	-	3013.75
metric	100.97*** (71)	18.92** (8)	0.067	.012	.900	.046	3021.20
scalar	113.44** (78)	12.75* (6)	0.069	.002	.882	.018	3018.84
	n (English) = 114; n (Arabic) = 75						
	Adjusted Covariate Model Socio-Demographic I						
configural	64.34*** (28)	-	0.111	-	.887	-	3322.65
configural modified	41.57* (26)		0.075		0.952		3298.36
metric	66.87** (33)	25.80**(7)	0.099	.024	0.895	.057	3310.24
scalar	72.67** (40)	6.14 (8)	0.088	-.011	0.898	-.003	3301.69
	n (English) = 130; n (Arabic) = 81						
	Adjusted Covariate Model Socio-Demographic II						
configural	44.03* (28)	-	0.085	-	.932	-	2478.16
metric	63.36** (35)	17.74** (8)	0.101	.016	.880	.052	2487.48
scalar	73.55** (42)	10.08 (8)	0.098	-.002	.866	-.014	2486.10
	n (English) = 93; n (Arabic) = 65						

*p < .05

**p < .01

***p < .001

Restricting factor loadings of indicators to being equal between the language groups significantly decreased model fit according to the change in all goodness of fit statistics, so that metric invariance was rejected as well. Due to its reasonable MI (greater than 3.84) [65], the loading of “autonomy” differed between the languages (Table 4). Restricting indicators’ intercepts to being equal between the language groups significantly decreased model fit according to the change of all goodness of fit statistics. So, if we assumed configural and metric invariance supported, scalar invariance had to be rejected. Modification indexes were significant (greater than 3.84) for four of seven thresholds (Table 5). The BIC values increased accordingly (Table 6) when restrictions were introduced, which supports the results obtained by the change of other fit indexes.

The results of the analyses with ordinal (ordered categorical) data with WLSMV estimator are reported in Suppl. Mat. 5, whereas configural, metric and scalar measurement invariance of the HSR instrument was either rejected or not evaluable due to the estimation problems.

We also conducted a robustness check accounting for sample clustering in the data. Only 18 persons were sharing a room (*n* = 9 rooms) and nesting in rooms is therefore rather negligible. The clustering effect of reception and accommodation centres on the CFA model for the responsiveness was controlled for by the two-level random intercept CFA analysis [55]. The results provide no significant intercept variance on the level of reception and accommodation centres (Var = 0.04, SE = 0.06). To additionally consider clustering when implementing MGCFA, we conducted the analyses using combined weights for the clustered data for rooms and facilities. The results were very similar to those obtained with data not weighted with respect to the model fit and its change (e.g., configural: CMIN _(df = 28)_ = 64, *p* < .001; RMSEA = 0.106; CFI = 0.841; BIC = 3672.13). Therefore, clustering effects did not change the results and we continued the remaining analyses using not weighted data.

Overall, we conclude that the measurement invariance of the HSR was rejected and therefore violated in our sample, whilst HSR did not exhibit configural measurement invariance. Assuming it nevertheless, metric and scalar measurement invariance were rejected as well.

### Non-parametric rescaling as basis of measurement invariance analysis

When using rescaled C variables (see Eq. 2) and the highest possible ratings, measurement invariance could not be improved, as compared with not rescaled data (Table 6). We do not provide further details for the model with highest possible ratings, i.e. parameters in Tables 4 and 5. However, with the lowest possible ratings, acceptable model fit was obtained for configural model according to CFI and RMSEA and there was no significant decrease in model fit statistics in the metric model. With respect to the scalar invariance, the change of CMIN was significant, the change of CFI was of a border value and the change of RMSEA was not significant. With the ordinal data analysis, metric and scalar measurement invariance were supported for this model (Suppl. Mat. 5), although the RMSEA value for the configural model was slightly above the benchmark value. Due to high and acceptable CFI, we accepted the configural categorical model. BIC provided no considerable change for metric and scalar models. The differences between the rescaled data with lowest vs. highest possible ratings in the case of disallocation of vignettes would be due to the little variability (SD ranged from 1.00 to 1.70) and extremity (Median = 7, Min = 1, Max = 7) of the values for the highest ratings. For the lowest possible ratings SD ranged from 2.20 to 2.60; the median varied between 1 and 2. Hence, the values of C for the highest or lowest rating may differ in other contexts, as it depends on the distribution of self-assesmmets. Overall, with non-parametric adjustment, improved measurement invariance analysis results were obtained at all levels. Particularly important is that the configural and metric measurement invariance was reached, which allowed for the evaluation of measurement invariance on different levels without restrictions.

### MGCFA covariate models with predictions from the parametric CHOPIT analysis

**Fig. 4:**
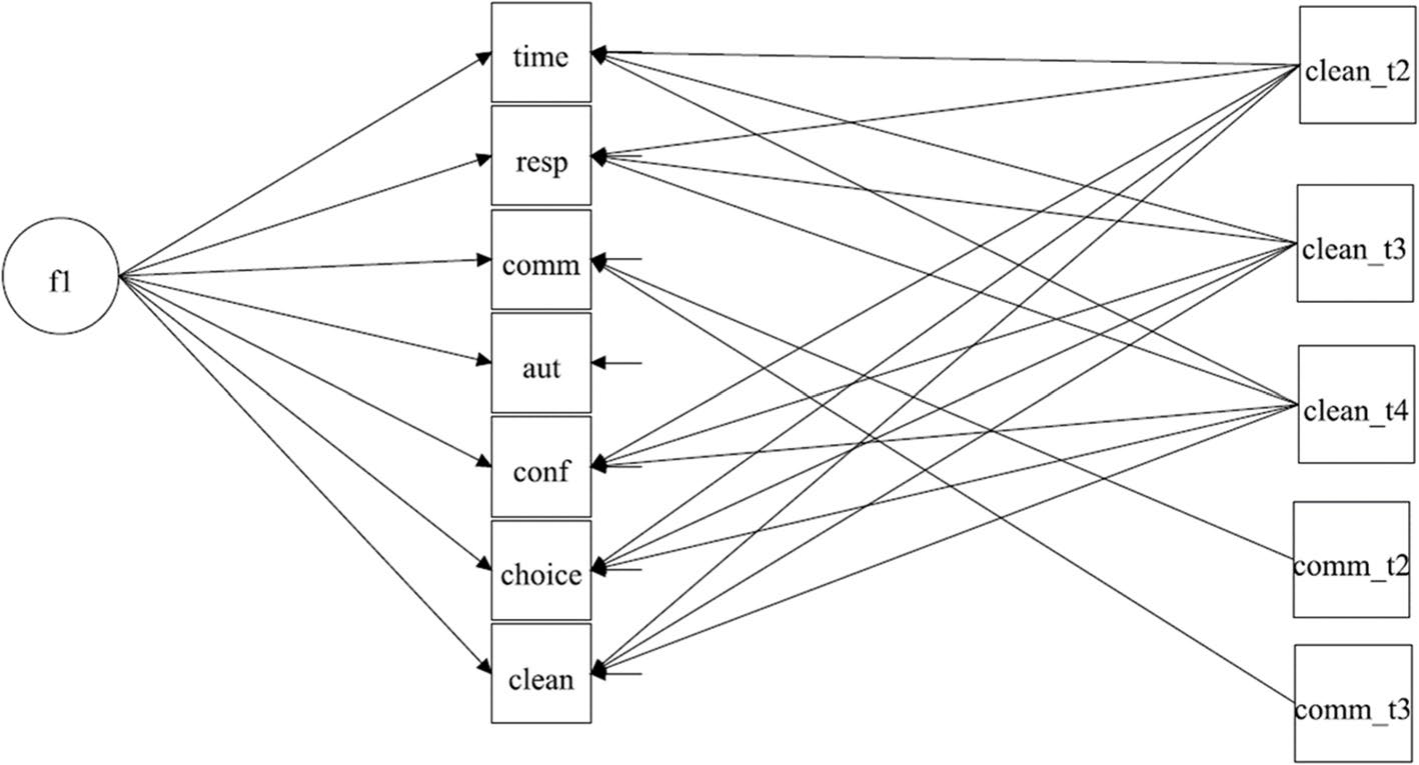
Covariate MGCFA Model of HSR with threshold values from the CHOPIT pediction Note. f1 factor HSR

As described in the data analysis section, we used vignette threshold values for the HSR indicators predicted by the CHOPIT analysis to evaluate how the parametric approach can be combined with measurement invariance analysis. If a threshold had a significant path (regression coefficient) to one or more manifest variables of HSR, it was included as a covariate variable in the MGCFA model. Significant paths on the HSR indicators were found and implemented in the final model for the quality of amenities vignettes (three threshold values) and for the communication vignettes (two threshold values) (Fig. 4; Mplus source code and output are included in Suppl. Mat. 4). Interestingly, threshold values from the quality of amenities vignettes correlated with most of the indicators of HSR in English and in Arabic (also with those with different content), except the autonomy indicator. Predicted thresholds from the communication vignettes correlated only with the communication self-assessments.

With the vignette thresholds as covariates, the configural model was associated with a tenable goodness-of-fit, so that configural invariance could be accepted (Table 6, Vignettes CHOPIT Thresholds). Metric invariance had to be rejected. With the analysis for ordinal (categorical) data, metric and scalar measurement invariance were supported by the BIC statistic (Suppl. Mat. 5). This means that including information on vignette evaluation in the models when utilizing the parametric approach allowed the acceptance of configural invariance. When using WLSMV estimator (that is treating data as ordered categorical), metric and scalar measurement invariance were supported as well.

### MGCFA covariate models with socio-demographic variables

**Fig. 5:**
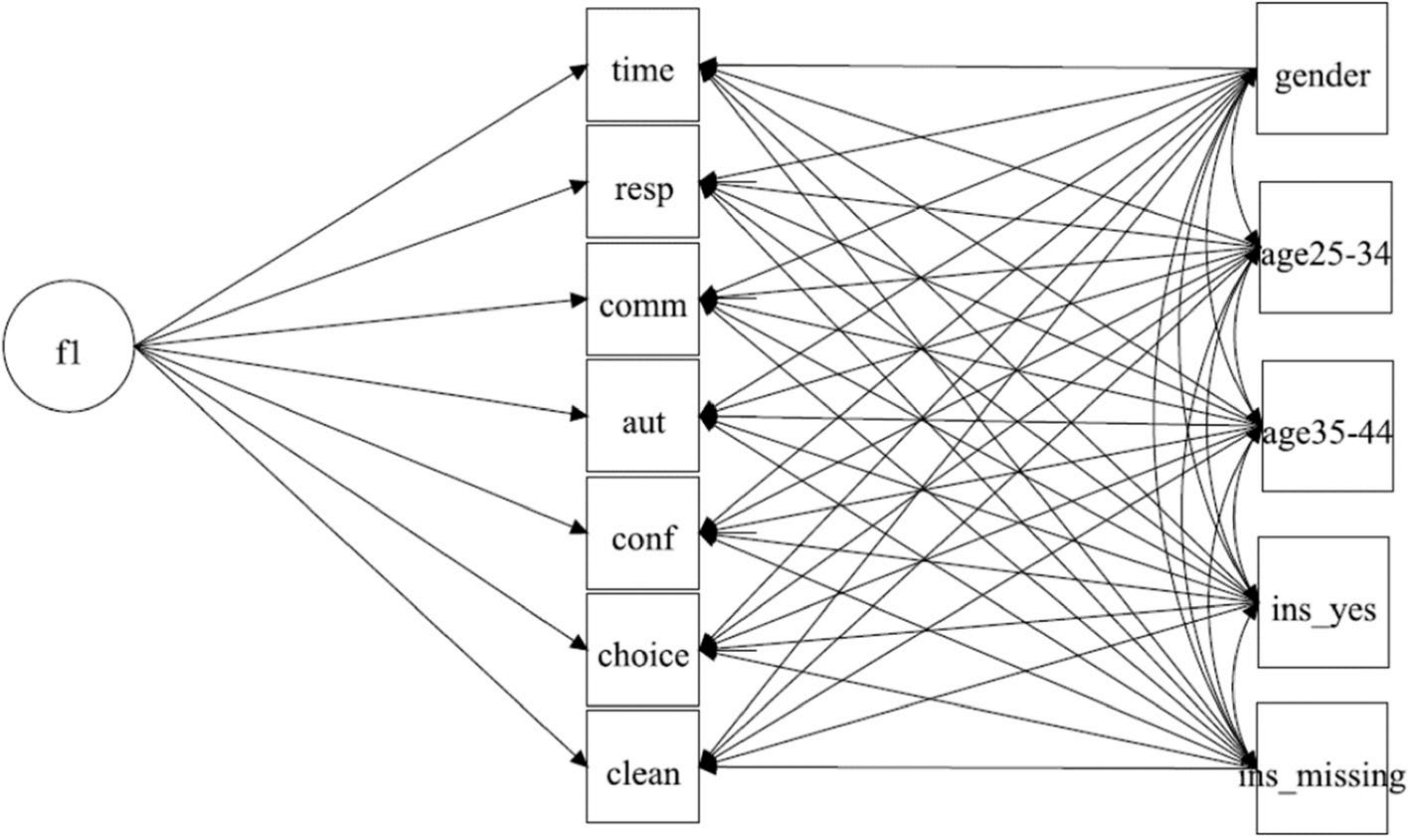
Covariate MGCFA Model of HSR with socio-demographic variables Note. f1 Factor HSR

In the last step, we included socio-demographic variables as covariates in the MGCFA analysis. When education was taken into account this markedly reduced sample size due to missing data and we conducted a separate analysis when education and gender were included as covariate variables. First, gender, age and possession of a health insurance card were regressed on each of the manifest variables of responsiveness (Fig. 5). This model did not provide a tenable model fit (Table 6, Socio-Demographic I), so that the configural invariance was rejected. Analyses for ordered categorical data (Suppl. Mat. 5) provided comparable results.

Second, we included gender and education as covariate variables in the last MCGFA model (Table 6, Socio-Demographic II). The configural model obtained a just acceptable model fit, but metric invariance was not supported. Therefore, socio-demographic covariates could not be used as mean of control of comparability bias.

### Summary and responding to the research questions

In response to the research question 1 that asked how does RC-DIF as evaluated with anchoring vignettes alter the measurement invariance, we state that considering RC-DIF in the analysis improved the results with respect to configural, metric and scalar measurement invariance.

Research question two issued differences between the implementation of parametric and non-parametric approaches. The results show that both approaches allow support for measurement invariance. Hence, with the non-parametric approach, a better configural model fit was obtained when using MLR and Pearson correlations in the MGCFA models to account for ordinality, whilst in the case of parametric approaches, treating data as ordered categorical and utilization of WLSMV estimator provided more satisfactory results.

In response to the third research question on whether comparable effects can be reached if consideration is taken of socio-demographic variables, which may be associated with the RC-DIF, we state that the improvement of the results of measurement invariance analysis is not given by the control of socio-demographic heterogeneity.

We conclude that RC-DIF was present in the data and negatively affected the results of measurement invariance analysis. Measurement invariance and therefore cross-cultural comparability could be improved when accounting for the RC-DIF, but not when accounting for socio-demographic heterogeneity.

## Discussion

We evaluated how to increase cross-cultural comparability in the data on HSR for Arabic- and English-speaking refugee groups. The cross-cultural comparability bias was evaluated by means of MGCFA measurement invariance analysis including different possibilities of control of RC-DIF. We compared the results of measurement invariance analysis when rescaling data or when including covariates produced with the help of anchoring vignettes. We also compared these possibilities with the inclusion of socio-demographic covariates in the models.

Configural, metric and scalar invariance of HSR between English and Arabic languages was initially violated, which allowed us to test several approaches to influence the non-satisfactory results of measurement invariance analysis. Here, data rescaling based on the implementation of anchoring vignettes provided satisfactory results and allowed configural, metric and scalar measurement invariance to be verified in the data. We also add to previous research [23, 25] to show that information from anchoring vignettes implemented in the MGCFA models has a strong and positive effect on the results of measurement invariance analysis. We add to previous research by evaluating comparability bias when using information gained from the parametric modelling approach for correction of RC-DIF by means of anchoring vignettes. We introduced a two-step procedure for this adjustment: 1) predict vignette threshold parameters from CHOPIT analysis and 2) introduce them into the MGCFA covariate models. However, different estimation methods (that is MLR or WLSMV) produced rather different results. With the WLSMV estimator, metric and scalar measurement invariance could be positively affected by non-parametric and parametric adjustment, whereas with the MLR configural measurement invariance could be consistently supported. More research is needed to evaluate these different methods when using anchoring vignettes.

Besides the use of anchoring vignettes, we included socio-demographic information on gender, age, education and health insurance in the measurement invariance analysis (this information was included in the parametric approach as well). This was not associated with an improved model fit (or bias reduction) with respect to configural and metric invariance. Unlike the CHOPIT-Analyses reported in the literature [19], we avoided using too many socio-demographic variables due to the small sample size and the homogeneity of our sample with respect to economic factors and living conditions. Further research can consider other and more socio-demographic variables when large sample sizes are used in order to investigate the possibility of their use when evaluating measurement invariance.

We conclude that RC-DIF was present in the HSR measurement. This violated all levels of measurement invariance between the evaluated English and Arabic speaking samples. We introduced modifications to achieve acceptable model fit for the configural model of HSR. The modification search we implemented means, however, that modified models should be validated in other samples. The results are therefore not generalizable to other data. By way of contrast, parametrically and non-parametrically re-scaled data enabled configural measurement invariance to be established, so that this data can be compared among the investigated languages without restrictions. Moreover, the models are generalizable to other samples, but the analyses without RC-DIF adjustment are not. Rescaling data or including covariates on the basis of anchoring vignettes could improve the cross-cultural comparability of our data, which supports the findings of previous studies [18, 23, 25]. The results also show that RC-DIF as assessed by anchoring vignettes is independent from the effect of socio-demographic variables on data comparability. We can therefore conclude that differences in cognitive response processes at the stage of response when using rating scales [32] account for a substantial bias associated with the rejection of configural, metric and scalar measurement invariance.

The improvement in measurement invariance results was obtained for non-parametric and parametric approaches to implement vignette data in self-evaluation data, although we were not able to implement the full set on vignettes for every indicator. The non-parametric approach used data on a selection of vignettes (bottom, middle and top) for every sampled person. The misallocation of the vignettes was given for approximately 30% of our data, which is disadvantageous due to lower clarity of the re-scaled variables and produced different plausible values for re-scaling for the corresponding respondents. Depending on the given self-assessments, the lower or the higher range of these values would be more valid. With anchoring vignettes of a higher quality and lower amount of misallocation of vignettes, the results of corrections would be more promising. Further research should address this issue. The parametric approach was based on predicted full vignette information from a respondents’ subgroup and not from the entire sample. However, for the parametric approach, we included information on vignettes from two indicators only (amenities and communication), because the vignette evaluations for other indicators did not correlate with any other self-evaluations. This might point to limited vignette consistency, as response patterns for vignettes and self-evaluations were different. Vignette consistency was given for vignette indicators we included into the modelling. The vignettes on indicator “amenities” not only exhibited consistency with the corresponding self-evaluations, but also with the self-evaluations of other HSR indicators. Therefore, for the control of RC-DIF in measurement invariance analysis, universal anchoring vignettes on topics other than self-assessments would work. Although we conducted analyses that do not rely on the assumption of vignette consistency and vignette equivalence, information gained from anchoring vignettes was useful in increasing model fit for measurement invariance analysis.

In our study, we used data from a unique population-based probabilistic refugee sample that allows for generalizability of results on the Arabic and English speaking refugee populations in the third largest German federal state. Therefore, we were able to replicate previous findings on the use of anchoring vignettes [25] for refugee sample and also considered the non-parametric approach. This was possible even though our sample was more heterogeneous than PISA samples used in previous research. The possibility of improving measurement invariance analysis results would be due to the high reliability of HSR in our data, obtained through a careful translation and cognitive pretesting of the instrument. This high reliability also allowed efficient MGCFA analyses despite small sample sizes [66]. However, to analyse the potential impact of education on measurement invariance and to implement the information in RC-DIF gained from the parametric approach more productively, replicating research with large samples should be conducted.

Finally, we only investigated exact measurement invariance analysis [4, 27], although less restrictive methods, such as alignment and a Bayesian approach are available [30, 31]. We did not use these methods due to their unresolved limitations. The alignment method is suitable in the case of large violations of measurement invariance for single items [31, 67] and has been found to be less sensitive in identifying non-comparability problems [28]. For the Bayesian method, prior information on invariance should be available [30], which was not the case in our research.

## Conclusions

Our study contributes to existing research on the comparability of health-related data and the methodology of measurement invariance analysis in several ways. We demonstrate that, in the context of studying a health concept, the implementation of anchoring vignettes can improve the comparability of statistical data in heterogeneous refugee populations. We further provide results that explain RC-DIF as a result of differences in the response process between individuals that use different languages. The adjustments for RC-DIF can therefore improve the results of measurement invariance analysis, which provides a solution to the problems of cross-cultural comparability in survey research [6, 68]. Use of full sets of anchoring vignettes is also associated with a higher burden on respondents, a longer survey time and increased research costs. Our experiences point to the possibilities for a more economic use of anchoring vignettes. This should be the focus of further research.

### Supplementary Information

**Additional file 1. **

## Data Availability

The dataset used during the current study is available from the corresponding author on reasonable request.

## Abbreviations

BIC: Bayesian Information Criterion
CFA: Conformatory Factor Analysis
CFI: Comparative Fit Index
CHOPIT: Hierarchical Ordered Regression Model
CMIN: Chi-square test
DIF: Differential Item Functioning
ESS: European Social Survey
GDPR: General Data Protection Regulations
HSR: Health System Responsiveness
IRT: Item Response Theory
ISSP: International Social Survey Program
LVM: Latent Variable Modelling
MANCOVA: Multivariate Analysis of Covariance
MGCFA: Multi-Group Confirmatory Factor Analysis
MI: Modification Indexes
MLR: Robust Maximum Likelihood Estimator
PISA: Programme for International Student Assessment
RC-DIF: Response-Category Differential Item Functioning
RMSEA: Root-Mean-Square Error of Approximation
SAGE: Global Ageing and Adult Health Survey
SEM: Structural Equation Modelling
SHARE: Survey of Health, Ageing and Retirement in Europe
TIMMS: Trends in International Mathematics and Science Study
WLSMV: Weighted Least Squares Estimator
WLS: Wisconsin longitudinal Study
WHO: World Health Organization
WHS: World Health Survey

## Footnotes

1 Additional types of measurement invariance exist, such as strict invariance that assumes equality in error variances across groups. We do not include it in our study because metric invariance is sufficient for the comparison of correlations and scalar invariance is sufficient for the comparison of means, which is also the aim of cross-cultural research. This is similar to the approach taken by other researchers [25, 26].
