# Supplementary material for: Ensuring cross-cultural data comparability by means of anchoring vignettes in heterogeneous refugee samples
Source: BMC Med Res Methodol. 2023 Sep 28;23:213. doi: 10.1186/s12874-023-02015-2 (PMC10536699; doi:10.1186/s12874-023-02015-2)
Supplement: Supplementary file 1 — Additional file 1. [file 12874_2023_2015_MOESM1_ESM.docx]

**Supplementary Material 1**

**Predicting C Variables (Non-Parametric Approach)**

See Wand, J., & King, G. (2007). Anchoring vignetttes in R: A (different kind of) vignette. Retrieved

from <http://wand.stanford.edu/anchors/doc/anchors.pdf>

# s_att to s_amn: Indicators of HSR; vg: top vignette, vm: medium vignetre, vs buttom vignette

atta <- anchors(s_att ~ vg+vm+vs, Data, method="C")

respa <- anchors(s_res ~ vg+vm+vs, Data, method="C")

coma <- anchors(s_com ~ vg+vm+vs, Data, method="C")

auta <- anchors(s_aut ~ vg+vm+vs, Data, method="C")

cona <- anchors(s_con ~ vg+vm+vs, Data, method="C")

choa <- anchors(s_cho ~ vg+vm+vs, Data, method="C")

amna <- anchors(s_amn ~ vg+vm+vs, Data, method="C")

## Saving adjusted values (insert + write.sav)

att <- insert(Data, atta, overwrite = TRUE)

resp <- insert(Data, respa, overwrite = TRUE)

com <- insert(Data, coma, overwrite = TRUE)

aut <- insert(Data, auta, overwrite = TRUE)

con<- insert(Data, cona, overwrite = TRUE)

cho<- insert(Data, choa, overwrite = TRUE)

amn<- insert(Data, amna, overwrite = TRUE)

## Saving Data to SPSS files

library (haven)

write_sav(time, " Respond_time.sav")

write_sav(resp, " Respond_resp.sav")

write_sav(com, " Respond_com.sav")

write_sav(aut, " Respond_aut.sav")

write_sav(con, " Respond_con.sav")

write_sav(cho, " Respond_cho.sav")

write_sav(amn, " Respond_amn.sav")

**Analysis Vignette Ordering**

voengl<-anchors.order(~vs+vm+vg, Data_engl)

summary(voengl,top=10,digits=3)

voarb<-anchors.order(~vs+vm+vg, Data_arabic)

summary(voarb,top=10,digits=3)

**Supplementary Material 2**

// ======================================

// CHOPIT ANALYIS

// ======================================

foreach n in com amn con cho res att aut { // com amn con cho res att aut

clear *

use respond.dta, clear

keep if speech<=1

drop if missing(s_`n', female, speech) // Missings

local i=0

foreach f in v_`n'_g v_`n'_mg v_`n'_m v_`n'_ms v_`n'_s s_`n' {

local ++i

rename `f' `n'`i' // rename vignettes & self reports

}

// reshape to long format

reshape long `n', i(id) j(item)

drop if `n'==0

drop if missing(`n')

tab item, gen(i)

rename i6 self

foreach g in female speech_2 age_2 age_3 age_4 ins_2 ins_3 {

gen s_`g' = self*`g'

}

gen vign = 1-self

// GLLAMM / CHOPIT - see Rabe-Hesketh & Skrondal 2003

eq thresh: female speech_2 age_2 age_3 age_4 ins_2 ins_3 // Threshold modell

eq het: vign self

constraint def 1 [lns1]self=0

eststo hopit_`n': ///

gllamm `n' s_female s_speech_2 s_age_2 s_age_3 s_age_4 s_ins_2 s_ins_3 ///

i1 i2 i3 i4 i5 ///

i(id) link(soprobit) s(het) ethresh(thresh) constr(1) init

// output simple Ordered Probit and CHOPIT model

esttab s_`n' hopit_`n' using `n'_hopit.rtf, replace nobasel label

*********

* predicted values answer category (via gllapred)

*********

help gllapred

foreach k in 1 2 3 4 {

gllapred vars`k', mu above(`k')

}

gen pr1_`n' = 1 - vars1

gen pr2_`n' = vars1 - vars2

gen pr3_`n' = vars2 - vars3

gen pr4_`n' = vars3 - vars4

gen pr5_`n' = vars4

//highest PP value

egen pr_`n'= rowmax(pr1 pr2 pr3 pr4 pr5)

// replace with ordinal answer value

replace pr_`n'=1 if pr_`n'==pr1_`n'

replace pr_`n'=2 if pr_`n'==pr2_`n'

replace pr_`n'=3 if pr_`n'==pr3_`n'

replace pr_`n'=4 if pr_`n'==pr4_`n'

replace pr_`n'=5 if pr_`n'==pr5_`n'

estimates dir hopit_*

mat list e(b)

*********

* save Coef results to locals

*********

local s_female = _b[s_female]

local s_speech_2 = _b[s_speech_2]

local s_age_2 = _b[s_age_2]

local s_age_3 = _b[s_age_3]

local s_age_4 = _b[s_age_4]

local s_ins_2 = _b[s_ins_2]

local s_ins_3 = _b[s_ins_3]

local _cut11_female = _b[_cut11:female]

local _cut11_speech_2 = _b[_cut11:speech_2]

local _cut11_age_2 = _b[_cut11:age_2]

local _cut11_age_3 = _b[_cut11:age_3]

local _cut11_age_4 = _b[_cut11:age_4]

local _cut11_ins_2 = _b[_cut11:ins_2]

local _cut11_ins_3 = _b[_cut11:ins_3]

local _cut11_cons = _b[_cut11:_cons]

di `_cut11_cons'

local _cut12_female = _b[_cut12:female]

local _cut12_speech_2 = _b[_cut12:speech_2]

local _cut12_age_2 = _b[_cut12:age_2]

local _cut12_age_3 = _b[_cut12:age_3]

local _cut12_age_4 = _b[_cut12:age_4]

local _cut12_ins_2 = _b[_cut12:ins_2]

local _cut12_ins_3 = _b[_cut12:ins_3]

local _cut12_cons = _b[_cut12:_cons]

di `_cut12_cons'

local _cut13_female = _b[_cut13:female]

local _cut13_speech_2 = _b[_cut13:speech_2]

local _cut13_age_2 = _b[_cut13:age_2]

local _cut13_age_3 = _b[_cut13:age_3]

local _cut13_age_4 = _b[_cut13:age_4]

local _cut13_ins_2 = _b[_cut13:ins_2]

local _cut13_ins_3 = _b[_cut13:ins_3]

local _cut13_cons = _b[_cut13:_cons]

di `_cut13_cons'

local _cut14_female = _b[_cut14:female]

local _cut14_speech_2 = _b[_cut14:speech_2]

local _cut14_age_2 = _b[_cut14:age_2]

local _cut14_age_3 = _b[_cut14:age_3]

local _cut14_age_4 = _b[_cut14:age_4]

local _cut14_ins_2 = _b[_cut14:ins_2]

local _cut14_ins_3 = _b[_cut14:ins_3]

local _cut14_cons = _b[_cut14:_cons]

di `_cut14_cons'

*********

* Thresholds

*********

gen tau1`n' = (`_cut11_cons' + ///

(`_cut11_female' * female) + ///

(`_cut11_speech_2' * speech_2) + ///

(`_cut11_age_2' * age_2) + ///

(`_cut11_age_3' * age_3) + ///

(`_cut11_age_4' * age_4) + ///

(`_cut11_ins_2' * ins_2) + ///

(`_cut11_ins_2' * ins_3))

gen tau2`n' = tau1`n' + ///

exp( `_cut12_cons' + ///

(`_cut12_female' * female) + ///

(`_cut12_speech_2' * speech_2) + ///

(`_cut12_age_2' * age_2) + ///

(`_cut12_age_3' * age_3) + ///

(`_cut12_age_4' * age_4) + ///

(`_cut12_ins_2' * ins_2) + ///

(`_cut12_ins_2' * ins_3))

gen tau3`n' = tau2`n' + ///

exp(`_cut13_cons' + ///

(`_cut13_female' * female) + ///

(`_cut13_speech_2' * speech_2) + ///

(`_cut13_age_2' * age_2) + ///

(`_cut13_age_3' * age_3) + ///

(`_cut13_age_4' * age_4) + ///

(`_cut13_ins_2' * ins_2) + ///

(`_cut13_ins_2' * ins_3))

gen tau4`n' = tau3`n' + ///

exp( `_cut14_cons' + ///

(`_cut14_female' * female) + ///

(`_cut14_speech_2' * speech_2) + ///

(`_cut14_age_2' * age_2) + ///

(`_cut14_age_3' * age_3) + ///

(`_cut14_age_4' * age_4) + ///

(`_cut14_ins_2' * ins_2) + ///

(`_cut14_ins_2' * ins_3))

gen new_`n' = `s_female'*s_female + `s_speech_2'*s_speech_2 + `s_age_2'*s_age_2 ///

+ `s_age_3'*s_age_3 + `s_age_4'*s_age_4 + `s_ins_2'*s_ins_2 + `s_ins_3'*s_ins_3 ///

if self==1

gen new`n' = 1 if new_`n'<tau1`n'

replace new`n'=2 if new_`n'>tau1`n' & new_`n'<tau2`n'

replace new`n'=3 if new_`n'>tau2`n' & new_`n'<tau3`n'

replace new`n'=4 if new_`n'>tau3`n' & new_`n'<tau4`n'

replace new`n'=5 if new_`n'>tau4`n'

keep if self==1

gen dp1`n' = pr1_`n'-p1`n'

gen dp2`n' = pr2_`n'-p2`n'

gen dp3`n' = pr3_`n'-p3`n'

gen dp4`n' = pr4_`n'-p4`n'

gen dp5`n' = pr5_`n'-p5`n'

keep id `n' p1`n' p2`n' p3`n' p4`n' p5`n' ///

`n' pr_`n' ///

pr1_`n' pr2_`n' pr3_`n' pr4_`n' pr5_`n' ///

dp1`n' dp2`n' dp3`n' dp4`n' dp5`n' ///

tau1`n' tau2`n' tau3`n' tau4`n' ///

new`n' new_`n'

save `n'_.dta, replace

}

******************

* Merge results back to data

******************

use respond.dta, clear

foreach n in com amn con cho res att aut {

merge 1:1 id using `n'_.dta

rename _merge `n'_merge

label var `n' "Original Values | Missing from HOPIT"

label var tau1`n' "Threshold 1 HOPIT Model (varying over Groups)"

label var tau2`n' "Threshold 2 HOPIT Model (varying over Groups)"

label var tau3`n' "Threshold 3 HOPIT Model (varying over Groups)"

label var tau4`n' "Threshold 4 HOPIT Model (varying over Groups)"

}

save respond_hopit.dta, replace

**Supplementary Material 3**

**Results of CHOPIT-Analysis**

While with the non-parametric approach we looked at the deviations in evaluations of vignettes, with the parametric approach we would like also to demonstrate how potential RC-DIF may impact evaluation of the variables under investigation. To demonstrate the potential effect of the RC-DIF on the results of the analyses, we compare the scores of cleanliness of the HSR as predicted by the regular ordered probit regression with the results of the CHOPIT analysis that used information on the potential RC-DIF form the vignettes. The results for two models are shown in Table 4. In the regular ordered probit model, the language indicator shows a significant and positive association with the rating of the quality of amenities of visited health care institutions, while the gender coefficient is not significant. The interpretation of these results would mean that Arabic-speaking refugees rate the cleanliness of facilities higher than English-speaking refugees, while there is no difference in the assessments of women and men. When controlling for RC-DIF with the help of the anchoring vignettes using CHOPIT analysis, these two associations are inverted. It is not the affiliation with language group, but with gender, that makes a difference in the assessment of cleanliness. Taking a closer look at the separate models that define the group specific thresholds by regressing them on the same covariates as the main model (bottom part of Table 4), we can see that the gender coefficient (.829) for the first vignette threshold – that is between responses “very bad” and “bad” – shows women more likely to choose the former category over the latter than men. Likewise, inspecting the results for the second threshold we find evidence for a divergence between refugees of different language groups (-1.765), which implies that speakers of the Arabic language refrain more often from using the “bad” instead of the “moderate” category. For the other thresholds, we can see the effects of age and health insurance card. These results again show the presence of RC-DIF, which also influences the effects of predictors on the dependent variable, as has been demonstrated in past research (e.g. Rice et al., 2012).

[insert Table 4 about here]

Table 4

Ordered Probit and CHOPIT Regression of Quality of Basic Amenities Ratings on Gender, Language, Age and Health Insurance Electronic Card

|  | Ordered Probit | | CHOPIT | |
| --- | --- | --- | --- | --- |
|  | β | SE | β | SE |
| Female | 0.108 | (0.186) | 0.837^*^ | (0.344) |
| Arabic speaking | 0.637^***^ | (0.191) | 0.493 | (0.277) |
| Age 25-29 | -0.239 | (0.208) | 0.0453 | (0.276) |
| Age 31-37 | -0.269 | (0.225) | 0.0258 | (0.420) |
| Age 38+ | -0.171 | (0.262) | 0.125 | (0.362) |
| Insurance Card | -0.0158 | (0.183) | 0.146 | (0.291) |
| Insurance Card - missing | -0.290 | (0.259) | -1.125^*^ | (0.517) |
| Threshold 1 (very bad / bad) |  |  |  |  |
| Female |  |  | 0.829^**^ | (0.284) |
| Arabic speaking |  |  | 0.224 | (0.201) |
| Age 25-29 |  |  | -0.234 | (0.208) |
| Age 31-37 |  |  | -0.392 | (0.308) |
| Age 38+ |  |  | -0.231 | (0.249) |
| Insurance Card |  |  | 0.513^*^ | (0.253) |
| Insurance Card - missing |  |  | -0.712 | (0.411) |
| Constant | -1.698^***^ | (0.224) | -1.670^***^ | (0.273) |
| Threshold 2 (bad / moderate) |  |  |  |  |
| Female |  |  | -0.137 | (0.425) |
| Arabic speaking |  |  | -1.765^*^ | (0.724) |
| Age 25-29 |  |  | 0.839^**^ | (0.323) |
| Age 31-37 |  |  | 0.677 | (0.477) |
| Age 38+ |  |  | -0.219 | (0.822) |
| Insurance Card |  |  | -0.270 | (0.328) |
| Insurance Card - missing |  |  | -0.378 | (0.616) |
| Constant | -1.294^***^ | (0.203) | -0.816^**^ | (0.315) |
| Threshold 3 (moderate / good) |  |  |  |  |
| Female |  |  | 0.0298 | (0.421) |
| Arabic speaking |  |  | 0.557 | (0.351) |
| Age 25-29 |  |  | -0.162 | (0.384) |
| Age 31-37 |  |  | 0.267 | (0.539) |
| Age 38+ |  |  | 0.209 | (0.459) |
| Insurance Card |  |  | -0.493 | (0.407) |
| Insurance Card - missing |  |  | 0.442 | (0.462) |
| Constant | -0.923^***^ | (0.193) | -1.122^***^ | (0.316) |
| Threshold 4 (good / very good) |  |  |  |  |
| Female |  |  | -0.0912 | (0.238) |
| Arabic speaking |  |  | -0.214 | (0.209) |
| Age 25-29 |  |  | 0.477^*^ | (0.238) |
| Age 31-37 |  |  | 0.681^*^ | (0.272) |
| Age 38+ |  |  | 0.749^**^ | (0.263) |
| Insurance Card |  |  | -0.145 | (0.210) |
| Insurance Card - missing |  |  | -0.239 | (0.342) |
| Constant | 0.0354 | (0.186) | -0.310 | (0.209) |

**Supplementary Material 4**

1) Mplus Source for Modified Configural Model of HSR

TITLE: Model_resp. !Title of the model

DATA: FILE = Resp.dat; !Where is the data

VARIABLE: NAMES ARE a1 - a106; !Variables in the data set

GROUPING IS a30(0=0 1=1); ! Grouping variable: 0 = English, 1 = Arabic

USEVARIABLES a2-a8 a81-a83 a77 a78; !Variables used in the model; a2-a8: HSR

MISSING ALL (-99); ! Declaration of missing values

ANALYSIS:

ESTIMATOR = MLR;

MODEL:

F1 BY a2* a3-a8; !Measurement part: CFA of HSR

F1@1; !Factor variance set to 1;

[F1@0]; !Factor mean set to 1; necessary to compare intercepts

a3 with a2 (1); !Correlated error term between “respect” and “attention”; equal in two groups

a4 with a3 (2); ! Correlated error term between “respect” and “communication”; equal in two groups

Model 1: !Specification for the Model Arabic, Factor loadings and intercepts are !different

F1 BY a2* a3-a8;

[a2-a8];

OUTPUT: STANDARDIZED (STDYX);

MODINDICES (3.84);

2) Mplus Source Data for Covariate Model CHOPIT Thresholds

TITLE: Model_resp. !Title of the model

DATA: FILE = Resp.dat; !Where is the data

VARIABLE: NAMES ARE a1 - a106; !Variables in the data set

GROUPING IS a30(0=0 1=1); ! Grouping variable: 0 = English, 1 = Arabic

USEVARIABLES a2-a8 a81-a83 a77 a78; !Variables used in the model; a2-a8: HSR indicators; a81-a83 amenities vignettes thresholds; a 77 a78 communication vignette thresholds, obtained by GLAMM analysis, see Appendix 2

MISSING ALL (-99); ! Declaration of missing values

ANALYSIS:

ESTIMATOR = MLR;

MODEL:

F1 BY a2* a3-a8; !Measurement part: CFA of HSR

a2-a3 a6-a8 ON a81-a83;

a4 ON a77 a78; !Structural part: HSR indicators regressed on threshold values

F1@1; !Factor variance set to 1;

[F1@0]; !Factor mean set to 1; necessary to compare intercepts

Model 1: !Specification for the Model Arabic, Factor loadings and intercepts are !different

F1 BY a2* a3-a8;

[a2-a8];

OUTPUT: STANDARDIZED (STDYX);

MODINDICES (3.84);

3) Mplus Source Data for Covariate Model Socio-Demographic Variables

TITLE: Model_resp. !Title of the model

DATA: FILE = RespSD.dat; !Where is the data

VARIABLE: NAMES ARE a1 - a128; !Variables in the data set

GROUPING IS a30(0=0 1=1); ! Grouping variable: 0 = English, 1 = Arabic

USEVARIABLES a2-a8 a35 a122 a123 a127 a128; !a2-a8 HSR indicators; a35 gender; a123 age 25-34 years; a123 age 35-44 years; a127 insurance yeas; a128 insurance missing

MISSING ALL (-99);

ANALYSIS:

ESTIMATOR = MLR;

MODEL:

F1 BY a2* a3-a8; !Measurement part: CFA of HSR

a2-a8 ON a35 a122 a123 a127 a128; !Structural part indicators of HSR are regressed on socio-demographic variables

F1@1;

[F1@0];

a3 with a2 (1); !correlated error terms as model modification

a3 with a4 (2); !correlated error term as model modification

Model 1:

F1 BY a2* a3-a8;

F1@1;

[a2-a8];

OUTPUT: STANDARDIZED (STDYX);

MODINDICES (3.84);

**Supplementary Material 5**

**Measurement Invariance Analyses for Ordinal Data**

We conducted different analyses with specifying data as ordered categorical variables. First, we used the default for measurement invariance analysis of Mplus software with the specification of measurement invariance analysis in the ANALYSIS command (Muthén & Muthén, 2014). The configural model obtained a poor model fit according to CMIN and RMSEA and had therefore to be rejected (Table 1). Metric invariance model could not be evaluated due estimation problems. Alternatively, measurement invariance analysis for categorical data can be conducted by means of mixture modelling, which we implemented. For these analyses, only BIC statistics are available for the evaluation of model fit. Mixture modelling revealed similar results to the analyses with MLR reported in the main text (Table 6 in main text) and metric and scalar invariance of the initial ordinal model had to be rejected. When using rescaled data from anchoring vignettes, configural, metric and scalar invariance could not be rejected. Hence, RMSEA for the configural model was slightly over the benchmark value. Due to a high CFI value we accepted the configural non-parametrically re-scaled model. However, the results were more tenable in the n analysis when using MLR (Table 6, main text).

The default analysis of Mplus for measurement invariance analysis did not allow for covariates to be introduced into the models. The covariate models were evaluated using mixture modelling as well. Configural measurement invariance is improved in the models with predicted vignette thresholds and socio-demographic variables gender, age, and medical insurance card (Socio-Demographic I). According to the change in the BIC statistic, metric and scalar invariance hold in the model with CHOPT predictions. For socio-demographic covariates, the modelling results are strongly comparable to those obtained with the analyses reported in the main text.

We can therefore conclude that using anchoring vignettes produces even more satisfactory results with respect to the support of metric and scalar measurement invariance of HSR indicators. However, these analyses have the disadvantage of a limited number of statistics for comparison purposes. In addition, configural measurement invariance cannot be evaluated when using BIC.

Table 1

Ordered Categorical Measurement Invariance Analysis for HSR and Non-Parametric Adjustment

| model | *χ^2^(df)* | *Δχ^2^(df)* | *RMSEA* | *ΔRMSEA* | *CFI* | *ΔCFI* |
| --- | --- | --- | --- | --- | --- | --- |
| Initial |  |  |  |  |  |  |
| configural | 117.83*** (28) | - | 0.165 | - | .947 | - |
| metric | NA (estimation problems) | - |  |  |  |  |
| scalar | 178.64*** (54) | 75.74*** (26) | 0.140 | - | .927 | - |
| Non parametric highest ratings | | | | | | |
| configural | 50.79** (28) | - | .091 | - | .993 | - |
| metric | 60.52** (34) | 10.30 (6) | .089 | .003 | .992 | .001 |
| scalar | 71.14** (54) | 12.20 (6) | .089 | .000 | .990 | .002 |

Note. MGCFA Model with categorical indicators and MODEL = CONFIGURAL METRIC SCALAR in the ANALYSIS command. Estimator used is WLSMV; polychoric correlations; This analysis is not available for the models with covariates.

Table 2

Sample-Size Adjusted BIC for Measurement Invariance Analyses with Ordered Categorical Data from Mixture Modeling

| Models | Initial | Adjusted Covariate CHOPIT Thresholds | Adjusted Covariate Socio-Demographic I |
| --- | --- | --- | --- |
| Configural | 3481.16 | 2897.48 | 3206.92 |
| Metric | 3488.33 | 2902.11 | 3219.59 |
| Scalar | 3498.39 | 2906.14 | 3212.81 |

Note. BIC Differences ≥ 6 provide evidence for strong differences (Raftery, 1995)
